# Crystal structure of iso­butyl­ammonium hydrogen oxalate hemihydrate

**DOI:** 10.1107/S1600536814022697

**Published:** 2014-10-24

**Authors:** Błażej Dziuk, Bartosz Zarychta, Krzysztof Ejsmont

**Affiliations:** a Faculty of Chemistry, University of Opole, Oleska 48, 45-052 Opole, Poland

**Keywords:** crystal structure, iso­butyl­ammonium hydrogen oxalate hemihydrate, hydrated mol­ecular salt, hydrogen bonding, materials engineering

## Abstract

In the title hydrated mol­ecular salt, C_4_H_12_N^+^·C_2_HO_4_^−^·0.5H_2_O, the O atom of the water mol­ecule lies on a crystallographic twofold axis. The dihedral angle between the CO_2_ and CO_2_H planes of the anion is 18.47 (8)°. In the crystal, the anions are connected to each other by strong near-linear O—H⋯O hydrogen bonds. The water mol­ecules are located between the chains of anions and iso­butyl­amine cations; their O atoms participate as donors and acceptors, respectively, in O—H⋯O and N—H⋯O hydrogen bonds, which form channels (dimensions = 4.615 and 3.387 Å) arranged parallel to [010].

## Related literature   

Structure *versus* properties research is an important area in material engineering, see: Desiraju (2010 ▶, 2013 ▶). For the crystal structures of oxalic acid salts with aliphatic amines, see: Dziuk *et al.* (2014*a* ▶,*b* ▶); Braga *et al.* (2012 ▶); Ejsmont (2006 ▶, 2007 ▶)); Ejsmont & Zaleski (2006*a* ▶,*b* ▶); MacDonald *et al.* (2001 ▶). For the characteristic structural motifs in ammonium di­carboxyl­ate salts, see: Ali *et al.* (2012 ▶). For motifs of hydrogen bonds containing carboxylate anions, see: Rodríguez-Cuamatzi *et al.* (2005 ▶).
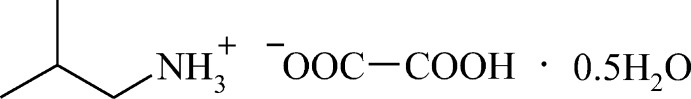


## Experimental   

### Crystal data  

2C_4_H_12_N^+^·2C_2_HO_4_^−^·H_2_O
*M* *_r_* = 344.36
Monoclinic, 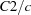

*a* = 21.2425 (9) Å
*b* = 5.6341 (1) Å
*c* = 16.5372 (6) Å
β = 119.141 (5)°
*V* = 1728.69 (10) Å^3^
*Z* = 4
Mo *K*α radiation
μ = 0.11 mm^−1^
*T* = 100 K
0.30 × 0.17 × 0.16 mm

### Data collection  

Oxford Diffraction Xcalibur diffractometer
5536 measured reflections
1696 independent reflections
1370 reflections with *I* > 2σ(*I*)
*R*_int_ = 0.019

### Refinement  

*R*[*F* ^2^ > 2σ(*F* ^2^)] = 0.028
*wR*(*F* ^2^) = 0.070
*S* = 1.03
1696 reflections
125 parameters
H atoms treated by a mixture of independent and constrained refinement
Δρ_max_ = 0.22 e Å^−3^
Δρ_min_ = −0.20 e Å^−3^

### 

Data collection: *CrysAlis CCD* (Oxford Diffraction, 2008 ▶); cell refinement: *CrysAlis RED* (Oxford Diffraction, 2008 ▶); data reduction: *CrysAlis RED*; program(s) used to solve structure: *SHELXS97* (Sheldrick, 2008 ▶); program(s) used to refine structure: *SHELXL97* (Sheldrick, 2008 ▶); molecular graphics: *SHELXTL* (Sheldrick, 2008 ▶); software used to prepare material for publication: *SHELXL97*.

## Supplementary Material

Crystal structure: contains datablock(s) global, I. DOI: 10.1107/S1600536814022697/hb7298sup1.cif

Structure factors: contains datablock(s) I. DOI: 10.1107/S1600536814022697/hb7298Isup2.hkl

Supporting information file. DOI: 10.1107/S1600536814022697/hb7298Isup3.cml

**Fig. 1.**
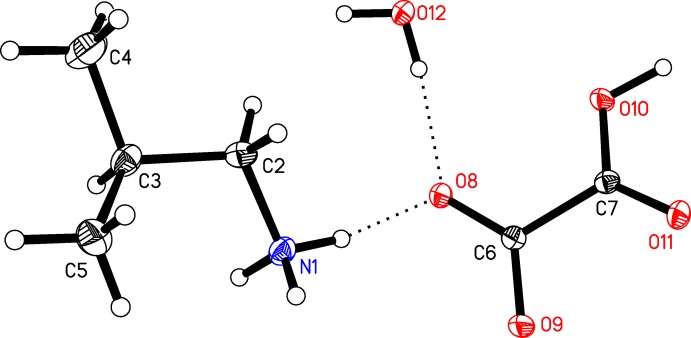
The molecular structure of (I), showing 50% displacement ellipsoids (arbitrary spheres for the H atoms). Hydrogen bonds are shown as dotted lines.

**Fig. 2.**
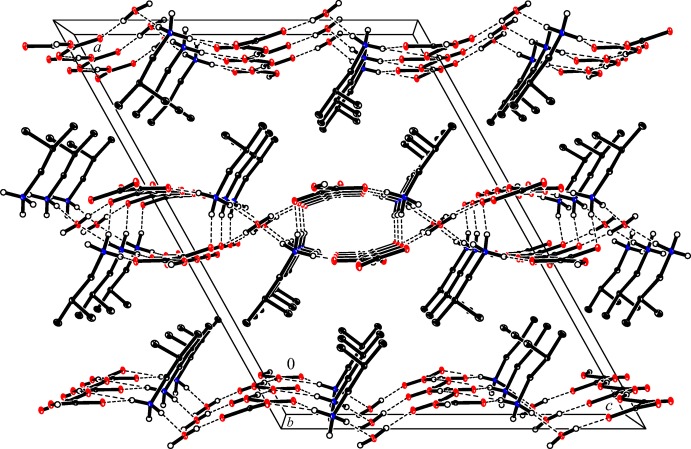
The packing diagram of (I), viewed along the b axis, showing the intermolecular hydrogen-bonding scheme (dashed lines).

. DOI: 10.1107/S1600536814022697/hb7298fig1.tif
The mol­ecular structure of (I), showing 50% displacement ellipsoids (arbitrary spheres for the H atoms). Hydrogen bonds are shown as dotted lines.

b . DOI: 10.1107/S1600536814022697/hb7298fig2.tif
The packing diagram of (I), viewed along the *b* axis, showing the inter­molecular hydrogen-bonding scheme (dashed lines).

CCDC reference: 1029482

Additional supporting information:  crystallographic information; 3D view; checkCIF report

## Figures and Tables

**Table 1:** Hydrogen-bond geometry (, )

*D*H*A*	*D*H	H*A*	*D* *A*	*D*H*A*
N1H1*A*O8	0.959(15)	1.963(15)	2.9111(13)	169.2(12)
N1H1*B*O9^i^	0.877(15)	2.031(15)	2.8333(13)	151.7(12)
N1H1*B*O11^i^	0.877(15)	2.518(14)	3.1968(13)	134.8(11)
N1H1*C*O12^ii^	0.940(15)	1.887(16)	2.8202(13)	171.4(13)
O12H12O8	0.853(15)	1.893(15)	2.7423(10)	173.0(16)
O10H10O9^iii^	0.988(17)	1.577(17)	2.5625(11)	175.3(17)

Symmetry codes: (i) 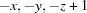; (ii) 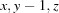; (iii) 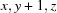.

## supplementary crystallographic information

### S1. Comment

Hydrogen bonds are very important in designing new materials. The structure *versus* properties research is important area in material engineering (Desiraju, 2010, 2013). Carboxylic acid molecules interact in crystals by strong hydrogen bonds, forming different motives *e.g.* isolated oxalate monoanions, dimers or as in the case of dicarboxylic acids, linear chains (Dziuk *et al.*, 2014*a*, 2014*b*; Braga *et al.*, 2012; Ejsmont & Zaleski 2006*a*, 2006*b*; Ejsmont, 2006, 2007). The crystal structure of the title salt, (I), consists of isobutylammonium cations, hydrogen oxalate anions and water molecules (Fig. 1). The Cambridge Structural Database (CSD; CONQUEST Version 1.16) has almost 70 structures of oxalic acid salts with aliphatic amines. The geometrical parameters of the isobutylammonium cation (Table 1) are comparable with those found in other crystal structures. The oxalate anions are connected to each other by strong O—H···O hydrogen bonds along the *b* axis. The isobutylammonium cations form N–H···O type HBs with the anions and water molecules. The O–H···O and N–H···O hydrogen bonds form channels (dimensions = 4.615 Å and 3.387 Å) arranged parallel to [010] direction (Fig. 2 and Table 2).

### S2. Experimental

Colourless prisms of (I) were grown at room temperature by slow evaporation of an aqueous solution containing isobutylamine and oxalic acid in a 1:1 stoichiometric ratio.

### S3. Refinement

All H atoms attached to atoms O and N were located in difference electron density maps and were freely refined with isotropic displacement factors [O–H = 0.853 (15) & 0.988 (17) and N–H = 0.877 (15) - 0.959 (15) Å]. The remaining H atoms were positioned geometrically and treated as riding on their parent C atoms, for methine group with distance of 0.98 Å and *U*_iso_ (H) = 1.2*U*_eq_(C), for methylene group with distance of 0.97 Å and *U*_iso_ (H) = 1.2*U*_eq_(C), for methyl group with distance of 0.96 Å and *U*_iso_ (H) = 1.5*U*_eq_(C), no refinement of their parameters.

### Crystal data

**Table:**

2C_4_H_12_N^+^·2C_2_HO_4_^−^·H_2_O	*F*(000) = 744
*M**_r_* = 344.36	*D*_x_ = 1.323 Mg m^−^^3^
Monoclinic, *C*2/*c*	Mo *K*α radiation, λ = 0.71073 Å
Hall symbol: -C 2yc	Cell parameters from 1696 reflections
*a* = 21.2425 (9) Å	θ = 3.8–26.0°
*b* = 5.6341 (1) Å	µ = 0.11 mm^−^^1^
*c* = 16.5372 (6) Å	*T* = 100 K
β = 119.141 (5)°	Prism, colourless
*V* = 1728.69 (10) Å^3^	0.30 × 0.17 × 0.16 mm
*Z* = 4	

### Data collection

**Table:**

Oxford Diffraction Xcalibur diffractometer	1370 reflections with *I* > 2σ(*I*)
Radiation source: fine-focus sealed tube	*R*_int_ = 0.019
Graphite monochromator	θ_max_ = 26.0°, θ_min_ = 3.8°
Detector resolution: 1024 x 1024 with blocks 2 x 2 pixels mm^-1^	*h* = −26→26
ω scan	*k* = −5→6
5536 measured reflections	*l* = −20→20
1696 independent reflections	

### Refinement

**Table:**

Refinement on *F*^2^	Primary atom site location: structure-invariant direct methods
Least-squares matrix: full	Secondary atom site location: difference Fourier map
*R*[*F*^2^ > 2σ(*F*^2^)] = 0.028	Hydrogen site location: inferred from neighbouring sites
*wR*(*F*^2^) = 0.070	H atoms treated by a mixture of independent and constrained refinement
*S* = 1.03	*w* = 1/[σ^2^(*F*_o_^2^) + (0.0414*P*)^2^ + 0.1361*P*] where *P* = (*F*_o_^2^ + 2*F*_c_^2^)/3
1696 reflections	(Δ/σ)_max_ < 0.001
125 parameters	Δρ_max_ = 0.22 e Å^−^^3^
0 restraints	Δρ_min_ = −0.20 e Å^−^^3^

### Special details

**Table:**

Geometry. All e.s.d.'s (except the e.s.d. in the dihedral angle between two l.s. planes) are estimated using the full covariance matrix. The cell e.s.d.'s are taken into account individually in the estimation of e.s.d.'s in distances, angles and torsion angles; correlations between e.s.d.'s in cell parameters are only used when they are defined by crystal symmetry. An approximate (isotropic) treatment of cell e.s.d.'s is used for estimating e.s.d.'s involving l.s. planes.
Refinement. Refinement of *F*^2^ against ALL reflections. The weighted *R*-factor *wR* and goodness of fit *S* are based on *F*^2^, conventional *R*-factors *R* are based on *F*, with *F* set to zero for negative *F*^2^. The threshold expression of *F*^2^ > σ(*F*^2^) is used only for calculating *R*-factors(gt) *etc*. and is not relevant to the choice of reflections for refinement. *R*-factors based on *F*^2^ are statistically about twice as large as those based on *F*, and *R*- factors based on ALL data will be even larger.

### Fractional atomic coordinates and isotropic or equivalent isotropic displacement parameters (Å^2^)

**Table:**

	*x*	*y*	*z*	*U*_iso_*/*U*_eq_	
N1	−0.06332 (5)	−0.08724 (18)	0.31560 (7)	0.0152 (2)	
H1A	−0.0236 (8)	0.019 (2)	0.3483 (9)	0.031 (4)*	
H1B	−0.0747 (8)	−0.146 (2)	0.3559 (10)	0.030 (4)*	
H1C	−0.0464 (8)	−0.208 (3)	0.2919 (10)	0.036 (4)*	
C2	−0.12455 (6)	0.0404 (2)	0.23817 (8)	0.0165 (3)	
H2A	−0.1056	0.1504	0.2102	0.020*	
H2B	−0.1500	0.1319	0.2628	0.020*	
C3	−0.17707 (6)	−0.1272 (2)	0.16433 (8)	0.0171 (3)	
H3A	−0.1503	−0.2241	0.1421	0.021*	
C4	−0.23158 (7)	0.0228 (2)	0.08384 (9)	0.0284 (3)	
H4A	−0.2066	0.1251	0.0625	0.043*	
H4B	−0.2589	0.1170	0.1041	0.043*	
H4C	−0.2635	−0.0794	0.0343	0.043*	
C5	−0.21430 (7)	−0.2922 (2)	0.20040 (9)	0.0247 (3)	
H5A	−0.1788	−0.3842	0.2509	0.037*	
H5B	−0.2461	−0.3966	0.1517	0.037*	
H5C	−0.2416	−0.2002	0.2214	0.037*	
C6	0.07522 (6)	0.27767 (19)	0.47629 (8)	0.0119 (2)	
C7	0.08118 (6)	0.52879 (19)	0.51722 (7)	0.0119 (2)	
O8	0.05406 (4)	0.25554 (13)	0.39204 (5)	0.0149 (2)	
O9	0.09169 (4)	0.11123 (13)	0.53399 (5)	0.0180 (2)	
O10	0.08298 (4)	0.69868 (14)	0.46405 (5)	0.0155 (2)	
H10	0.0884 (9)	0.855 (3)	0.4940 (12)	0.057 (5)*	
O11	0.08261 (4)	0.55763 (14)	0.59056 (5)	0.0169 (2)	
O12	0.0000	0.5785 (2)	0.2500	0.0165 (3)	
H12	0.0198 (9)	0.486 (3)	0.2967 (10)	0.048 (5)*	

### Atomic displacement parameters (Å^2^)

**Table:**

	*U*^11^	*U*^22^	*U*^33^	*U*^12^	*U*^13^	*U*^23^
N1	0.0170 (5)	0.0171 (5)	0.0115 (5)	−0.0011 (4)	0.0070 (4)	0.0001 (4)
C2	0.0180 (6)	0.0156 (6)	0.0159 (6)	0.0012 (5)	0.0082 (5)	0.0039 (5)
C3	0.0170 (6)	0.0213 (6)	0.0130 (6)	0.0028 (5)	0.0073 (5)	−0.0003 (5)
C4	0.0218 (7)	0.0356 (8)	0.0207 (7)	0.0002 (6)	0.0048 (6)	0.0079 (6)
C5	0.0238 (7)	0.0195 (7)	0.0256 (7)	−0.0044 (5)	0.0080 (6)	0.0005 (5)
C6	0.0120 (6)	0.0123 (6)	0.0127 (6)	0.0000 (4)	0.0072 (5)	0.0004 (4)
C7	0.0098 (6)	0.0131 (6)	0.0117 (5)	0.0001 (4)	0.0043 (4)	0.0010 (4)
O8	0.0216 (4)	0.0129 (4)	0.0110 (4)	0.0002 (3)	0.0087 (4)	−0.0010 (3)
O9	0.0304 (5)	0.0111 (4)	0.0140 (4)	0.0002 (3)	0.0119 (4)	0.0015 (3)
O10	0.0248 (5)	0.0097 (4)	0.0137 (4)	−0.0006 (3)	0.0107 (4)	0.0008 (3)
O11	0.0252 (5)	0.0156 (4)	0.0119 (4)	−0.0009 (3)	0.0106 (4)	−0.0016 (3)
O12	0.0227 (7)	0.0142 (6)	0.0103 (6)	0.000	0.0062 (5)	0.000

### Geometric parameters (Å, º)

**Table:**

N1—C2	1.4921 (14)	C4—H4C	0.9600
N1—H1A	0.959 (15)	C5—H5A	0.9600
N1—H1B	0.877 (15)	C5—H5B	0.9600
N1—H1C	0.940 (15)	C5—H5C	0.9600
C2—C3	1.5167 (16)	C6—O8	1.2445 (13)
C2—H2A	0.9700	C6—O9	1.2599 (13)
C2—H2B	0.9700	C6—C7	1.5468 (15)
C3—C5	1.5181 (17)	C7—O11	1.2092 (13)
C3—C4	1.5249 (16)	C7—O10	1.3128 (13)
C3—H3A	0.9800	O10—H10	0.988 (17)
C4—H4A	0.9600	O12—H12	0.853 (15)
C4—H4B	0.9600		
			
C2—N1—H1A	110.0 (8)	C3—C4—H4B	109.5
C2—N1—H1B	112.6 (9)	H4A—C4—H4B	109.5
H1A—N1—H1B	107.4 (12)	C3—C4—H4C	109.5
C2—N1—H1C	110.0 (9)	H4A—C4—H4C	109.5
H1A—N1—H1C	106.2 (12)	H4B—C4—H4C	109.5
H1B—N1—H1C	110.4 (12)	C3—C5—H5A	109.5
N1—C2—C3	112.53 (9)	C3—C5—H5B	109.5
N1—C2—H2A	109.1	H5A—C5—H5B	109.5
C3—C2—H2A	109.1	C3—C5—H5C	109.5
N1—C2—H2B	109.1	H5A—C5—H5C	109.5
C3—C2—H2B	109.1	H5B—C5—H5C	109.5
H2A—C2—H2B	107.8	O8—C6—O9	126.09 (10)
C2—C3—C5	112.62 (9)	O8—C6—C7	119.31 (9)
C2—C3—C4	107.83 (10)	O9—C6—C7	114.58 (9)
C5—C3—C4	111.28 (10)	O11—C7—O10	125.39 (10)
C2—C3—H3A	108.3	O11—C7—C6	121.24 (10)
C5—C3—H3A	108.3	O10—C7—C6	113.37 (9)
C4—C3—H3A	108.3	C7—O10—H10	110.3 (10)
C3—C4—H4A	109.5		
			
N1—C2—C3—C5	63.80 (13)	O9—C6—C7—O11	−18.47 (15)
N1—C2—C3—C4	−173.04 (10)	O8—C6—C7—O10	−18.64 (14)
O8—C6—C7—O11	160.16 (10)	O9—C6—C7—O10	162.72 (9)

### Hydrogen-bond geometry (Å, º)

**Table:**

*D*—H···*A*	*D*—H	H···*A*	*D*···*A*	*D*—H···*A*
N1—H1*A*···O8	0.959 (15)	1.963 (15)	2.9111 (13)	169.2 (12)
N1—H1*B*···O9^i^	0.877 (15)	2.031 (15)	2.8333 (13)	151.7 (12)
N1—H1*B*···O11^i^	0.877 (15)	2.518 (14)	3.1968 (13)	134.8 (11)
N1—H1*C*···O12^ii^	0.940 (15)	1.887 (16)	2.8202 (13)	171.4 (13)
O12—H12···O8	0.853 (15)	1.893 (15)	2.7423 (10)	173.0 (16)
O10—H10···O9^iii^	0.988 (17)	1.577 (17)	2.5625 (11)	175.3 (17)

Symmetry codes: (i) −*x*, −*y*, −*z*+1; (ii) *x*, *y*−1, *z*; (iii) *x*, *y*+1, *z*.
